# A Simple Culture Method Enhances the Recovery of Culturable Actinobacteria From Coastal Sediments

**DOI:** 10.3389/fmicb.2021.675048

**Published:** 2021-06-14

**Authors:** Zhaobin Huang, Shiqing Mo, Lifei Yan, Xiaomei Wei, Yuanyuan Huang, Lizhen Zhang, Shuhui Zhang, Jianzong Liu, Qingqing Xiao, Hong Lin, Yu Guo

**Affiliations:** ^1^College of Oceanology and Food Science, Quanzhou Normal University, Quanzhou, China; ^2^Fujian Province Key Laboratory for the Development of Bioactive Material From Marine Algae, Quanzhou, China

**Keywords:** culturable bacteria, water extraction, coastal sediments, 16S rRNA gene, amplicon sequence variant, culturable actinobacteria

## Abstract

Molecular methods revealed that the majority of microbes in natural environments remains uncultivated. To fully understand the physiological and metabolic characteristics of microbes, however, culturing is still critical for microbial studies. Here, we used bacterial community analysis and four culture media, namely, traditional marine broth 2216 (MB), water extracted matter (WEM), methanol extracted matter (MEM), and starch casein agar (SCA), to investigate the diversity of cultivated bacteria in coastal sediments. A total of 1,036 isolates were obtained in pure culture, and they were classified into five groups, namely, Alphaproteobacteria (52.51%), Gammaproteobacteria (23.26%), Actinobacteria (13.32%), Firmicutes, and Bacteroidetes. Compared to other three media, WEM recovered a high diversity of actinobacteria (42 of 63 genotypes), with *Micromonospora* and *Streptomyces* as the most cultivated genera. Amplicon sequencing of the bacterial 16S ribosomal RNA (rRNA) gene V3–V4 fragment revealed eight dominant groups, Alphaproteobacteria (12.81%), Gammaproteobacteria (20.07%), Deltaproteobacteria (12.95%), Chloroflexi (13.09%), Bacteroidetes (8.28%), Actinobacteria (7.34%), Cyanobacteria (6.20%), and Acidobacteria (5.71%). The dominant members affiliated to Actinobacteria belonged to “*Candidatus* Actinomarinales,” “*Candidatus* Microtrichales,” and Nitriliruptorales. The cultivated actinobacteria accounted for a small proportion (<5%) compared to the actinobacterial community, which supported that the majority of actinobacteria are still waiting for cultivation. Our study concluded that WEM could be a useful and simple culture medium that enhanced the recovery of culturable actinobacteria from coastal sediments.

## Introduction

Coastal sediments are inhabited by diverse and abundant microbes, which function as key drivers in biogeochemical processes of element cycling and organic matter decomposition (Bertics and Ziebis, 2009; Anantharaman et al., 2016; Dyksma et al., 2016). They are also a source for discovery of marine microbial-derived reference pharmaceuticals and industrial enzymes (Bull et al., 2005; Jose and Jha, 2017). Molecular tools including next generation sequencing (NGS) of microbial DNA sequences, such as genomics and metagenomics, improved our knowledge on microbial diversity, genomic features, and potential metabolic functions inferred from gene annotation (Dyksma et al., 2016). In addition, genomics and metagenomics strengthened the phylogenetic placement of uncultivated bacteria from the natural environment through comparison of genomic relatedness and phylogenomic reconstruction (Yarza et al., 2014; Parks et al., 2018). Although culture-dependent method captured the bacteria with extremely low abundance (rare bacteria) (Shade et al., 2012), to gain deep insights into the physiological, metabolic, and functional role of environmental microorganisms, however, culturing microbes in the laboratory is still necessary for microbial studies (Zengler et al., 2002; Leadbetter, 2003). First, bringing bacteria into culture provides a platform for experimental testing of their physiological and metabolic function in element cycling compared to stable isotope probing (SIP) (Neufeld et al., 2007) or bioorthogonal non-canonical amino acid tagging (BONCAT; Hatzenpichler et al., 2016), which was mainly used in the study of uncultivated bacteria *in situ*. Second, pure cultures are needed for species delineation for microbial taxonomy based on physiological and biochemical tests. Third, cultures can be explored for the discovery of novel natural product, such as antibiotics, for molecular design of biosynthetic elements (Joint et al., 2010), and for the potential application of the isolates to bioremediation and environmental protection. For example, survey of the bacterial 16S ribosomal RNA (rRNA) gene by NGS revealed a dominant and ubiquitous core group named “JTB255-Marine Benthic Group (MBG)” (now classified into the order Woeseiales) in coastal sediments (Dyksma et al., 2016). Isolation and cultivation of the representative species *Woeseia oceani* of MBG expanded our understanding of physiological and metabolic features with heterotrophy and facultative autotrophy that was deduced from genomic repertoire of uncultivated members by NGS (Du et al., 2016). In addition, a myriad of biologically active compounds were discovered from the culturable actinobacteria, which were isolated from sediment, invertebrate symbionts, and other marine environments, such as the genera *Streptomyces* (Bull and Stach, 2007) and *Micromonospora* (Qi et al., 2020). Turbinmicin, a novel promising compound produced by the marine ascidian-associated *Micromonspora* sp. WMMC-414, showed potent efficacy against the multidrug-resistant fungi *Candia auris* (Zhang et al., 2020).

Several factors can influence the bacterial cultivability, including the substrates (electron donors and acceptors) and growth conditions, cell dormancy (persisting cells), cell interdependency, and so on (Lewis et al., 2021). The nutrient composition of culture media is a major factor affecting the recovery of bacteria from natural environments (Connon and Giovannoni, 2002). Marine agar 2216 (BD) or marine broth 2216 (BD) are frequently and widely used in the isolation of marine bacteria from various habitats, including seawater, sediment, or the isolation of marine bacteria associated with animals or plants (Gram et al., 2010; Sanz-Sáez et al., 2020). Several studies revealed that adding extracted inorganic compounds from the natural environment as nutrient sources could be an effective method to isolate novel bacteria from soil (Nguyen et al., 2018) and groundwater (Wu et al., 2020). For example, soil nutrients extracted by autoclaving an equal mixture of water and soil were found to give higher numbers of colonies (Taylor, 1951a). Organic matter extracted from soil using NaOH facilitated the isolation of novel bacterial strains that could not grow on conventional media (Hamaki et al., 2005). Organic matter extracted from soil using 80% methanol enabled the cultivation of a large number of isolates, representing new taxa that were previously uncultured (Nguyen et al., 2018).

Quanzhou Bay is located in southeast Fujian Province, China, with a flat tidal area of 89.8 km^2^ (Lin et al., 2019), and is a typical estuarine ecosystem with two rivers (Jinjiang River and Luoyangjiang River) run into the bay (Supplementary Figure 1). It is reported that Quanzhou Bay has the largest number of pollutant load in the estuaries of Fujian Province, with relatively high concentration of N and P (Cui et al., 2011). The major habitats found in Quanzhou Bay are *Spartina alterniflora* growing area, oyster and/or razor clam farming area, and mangrove growing area (Supplementary Figure 1). The mangrove growing area in Quanzhou Bay has become one of the largest restored mangrove preservation areas in China. The biological functions in these habitats may result from microbial diversity and activity. So far, there are few reports of bacterial cultivation from the coastal sediments in Quanzhou Bay (Hu et al., 2020; Huang et al., 2020).

In this study, four cultures media were used to investigate the diversity of cultivated bacteria from coastal sediments, namely, traditional marine broth 2216 (MB), a medium based on aqueous extracts of sediment as the sole source of nutrients (water extracted matter, WEM), a medium based on methanolic extracts of sediment as the sole source of nutrients (methanol extracted matter, MEM), and starch casein agar (SCA). The two media, WEM and MEM, are less used in the campaign of bacterial cultivation of coastal sediment samples. We tested sediments of the three coastal habitats, a *S. alterniflora* growing habitat, an oyster farming habitat, and a mangrove habitat in Quanzhou Bay. A large number of bacterial strains were obtained, and these were taxonomically characterized based on nearly full-length 16S rRNA gene sequencing. To evaluate the bacterial cultivability, bacterial community structure of the sediment samples from the three habitats was determined using MiSeq amplicon sequencing of the V3–V4 regions of the 16S rRNA gene.

## Materials and Methods

### Sediment Sampling

Surface sediment samples (∼5 cm) were collected from three distinct habitats located in Quanzhou Bay, Fujian province, China, during the period of November 2018 to September 2019 and used for culture-dependent studies. The three habitats include an *S. alterniflora* growing habitat, an oyster farming habitat, and a mangrove habitat (Supplementary Figure 1 and Supplementary Table 1). Two or three sediment samples of each habitat were collected and put into sterile packages and taken to the laboratory and processed immediately to prepare the culture media. The samples were put in cool temperature for 3 days before streaking on the culture plates. For bacterial 16S rRNA gene amplicon sequencing, 32 sediment samples from the above three habitats were collected during the period of July 2019 to November 2020. The samples were put in −20°C for until DNA extraction.

### Culture Media

Four culture media were used for the isolation of bacteria from the coastal sediments, namely, MB, WEM, MEM, and SCA. MB consisted of 3.7% marine broth 2216 (BD) and 1.5% agar (BD), which were widely used in cultivation of marine bacteria (Sanz-Sáez et al., 2020). WEM resembled the soil extract using water (Taylor, 1951b; Nguyen et al., 2018): 200 g sediment was extracted with an equal volume of pure water by shaking (160 rpm) overnight at 30°C, the water-sediment slurry was centrifuged at 3,000 rpm for 10 min, and the supernatant was cleared by filtration through filter paper (d = 15 cm). The filtrate was supplemented with 1.5% agar (BD) and then sterilized. The pH and salinity of this medium was 6.5–7.0% and 0.5–0.8% (Supplementary Table 1). MEM was prepared following the previous study (Nguyen et al., 2018): 200 g sediment was extracted with an equal volume of 80% methanol (AR) by shaking (160 rpm) overnight at 30°C, the slurry was cleared by filtration through filter paper as described above, another 200-mL portion of 80% methanol was added to the slurry, and filtration was repeated. The filtrates were pooled and evaporated in a water bath of 37°C. The residue was dissolved in 50 ml pure water and sterilized by filtration through a 0.22-μm pore size membrane filter (Millipore). The medium was prepared with mixing half strength artificial seawater and 2.5% methanol extracted matter, and 1.5% agar (BD), followed by autoclaving sterilization. SCA contained 10 g/L starch, 0.3 g/L casein, and 1.5% agar (BD) and dissolved in coastal seawater/pure water (1:1, by volume), which was often used in actinobacteria from marine samples (Bukhari et al., 2013; Salim et al., 2017).

### Culturing and Bacterial Isolation

Sediment samples of 0.2 g were serially diluted in 0.9 mL sterile natural seawater (sterilization of 121°C for 20 min), and 0.1-mL portions of the dilutions were spread onto the four culture media plates described above. The plates were incubated at 28–30°C for 30 days. The colonies were picked in the process of incubation (note: we tried to pick all of the colonies from one plate but missed the colonies with small size) and streaked onto MB agar twice to obtain pure isolates and recover enough cell biomass for preservation and identification. The isolates were inoculated in 30 mL MB using a 100-mL glass flask at 30°C for shaking for 3–7 days to obtain cell biomass, and the cells were preserved in 20% glycerol (v/v) at −80°C.

### DNA Extraction, PCR Amplification, and Sequencing of 16S rRNA Gene

For bacterial community analysis, the total DNA of 0.3–0.5 g sediment samples were extracted using FastDNA Spin Kit for Soil (MP Bio, CA, United States) following the manufacturer’s manual. The quality of total DNA was controlled using 1% agarose electrophoresis, and the purity (the values of OD260/280 and OD260/230) and the concentration were measured using NanoDrop 2000 (Thermo Scientific, Waltham, MA, United States).

The bacterial 16S rRNA gene V3–V4 fragment was amplified using universal primers 338F (5′-ACTCCTACG GGAGGCAGCAG-3′) and 806R (5′-GGACTACHVGGG TWTCTAAT-3′) (Huws et al., 2007; Caporaso et al., 2011). The PCR reactions contained 4 μL 5× FastPfu buffer, 2 μL 2.5 mM deoxyribonucleotide triphosphates (dNTPs), 0.8 μL primers each (5 μM), 0.4 μL Fast*Pfu* polymerase, and 10 ng total DNA. The PCR program contained denaturation at 95°C for 3 min, 27 cycles of 95°C for 30 s–55°C for 30 s–72°C for 45 s, and extension at 72°C for 10 min. MiSeq sequencing was carried out by adding dual barcode following the Illumina manufacturer’s instruction (Shanghai Majorbio Bio-Pharm Technology Co., Ltd., Shanghai, China).

For the cultivated isolates, cells were collected from liquid cultures by centrifugation at 13,000 rpm for 1 min and were used for genomic DNA extraction using a Bacterial DNA Extraction Kit (Shanghai SBS, Biotech Co., Shanghai, China), following the manufacturer’s manual.

The 16S rRNA gene sequences were amplified using PCR with bacterial primers Eubac27F (5′-AGAGTTTGATCCT GGCTCAG-3′) and 1492R (5′-GGTTACCTTGTTACGACTT-3′) (DeLong, 1992). The PCR reactions were carried out in 50-μl systems, which contained 5 μL 10× *Ex* buffer, 2 μL of each primer (10 mM), 4 μL dNTP (2.5 mM), 0.25 μL *Ex* Taq (5 U/μL, TaKaRa), and 50–100 ng DNA. The PCR program consisted of denaturation at 94°C for 5 min, 30 cycles of 94°C for 45 s–55°C for 45 s–72°C for 1 min, and extension at 72°C for 10 min. PCR products were separated using 1.5% agarose electrophoresis. The nearly full-length sequences of the 16S rRNA gene were obtained using Sanger sequencing on ABI 3730 equipment (Shanghai Majorbio Bio-Pharm Technology Co., Ltd., Shanghai, China). The two fragments were assembled into the nearly full-length 16S rRNA gene sequence using DNAMAN v. 7.0 with sequence assembly, and base quality of the starting and end bases was checked according to the chromatogram using Chromas v. 1.62 program.

### Amplicon Sequence Variants Analysis and Identification of Cultivated Isolates

Bioinformatic processing of 16S rRNA gene amplicons was carried out using the protocol for bacterial 16S rRNA community of water and sediments described by von Hoyningen-Huene et al. (2019) with the following modification. The paired-end (PE) reads from the Illumina MiSeq sequencing were first quality checked using fastp (Chen et al., 2018) with the quality phred score of 20 (−q 20), minimum read length of 200 (−l 200), base correction by overlap (−c), as well as 5′-end trimming (−5 4) and 3′-end trimming (−3 4). Then, the barcodes and primers were clipped using cutadapt (version 1.9.1) with default settings and further assembled using PEAR (version 0.9.2). The assembled sequences were then sorted by length (–minseqlength 390, –maxseqlength 450), dereplicated (–derep_fulllength), and denoised with UNOISE3 (–cluster_unoise–minsize 4) using vsearch (Rognes et al., 2016) to produce the amplicon sequence variants (ASVs). The taxonomy of each ASV was assigned using blast+ against SILVA SSU 132 database and classified into different taxonomic levels using the lowest common ancestor (LCA) method in MEGAN community Edition (version 6.11.1) (Huson et al., 2016).

The 16S rRNA gene sequences of the isolates were clustered at 99% sequence similarity using vsearch (Rognes et al., 2016) to define the genotype. The taxonomic assignment was performed by using the RDP Release 11 (Ribosomal Data Project) classifier tool (Wang et al., 2007). The representative species were searched against the SILVA SSU database (release 132) using the blast+ program (Camacho et al., 2009) and taxonomically classified into genus level using the LCA method in MEGAN (Huson et al., 2016). The diversity indices (Shannon and Evenness) were calculated using Plymouth Routines in Multivariate Ecological Research (Primer 5, v. 5.2.9).

Sequence similarities of representative genotypes were compared using three reference databases, namely, SILVA SSU v. 132 database, RDP database, and EzBioCloud database (2018.11) using the blast+ tools (Camacho et al., 2009) and the nucleotide database in National Center for Biotechnology Information (NCBI).

### Phylogenetic Analysis of Isolates

Reference sequences were retrieved from SILVA SSU v. 132 database using blast+ with e-value of 1e-5 (Camacho et al., 2009). Multiple sequences were aligned using muscle v. 3.8.31. Maximum-likelihood phylogenetic trees were inferred using Raxml (Stamatakis, 2015) with the following parameters: p = 12,345, x = 12,345 with the bootstrap value of 100. The tree was edited using iTOL (Letunic and Bork, 2007).

### Availability of Nucleotide Sequences

The 16S rRNA gene sequences of the isolates determined in this study were deposited in GenBank under the accession numbers MT829339-MT829543, MK881086, MK791303, MN850335, MN908333, MN227660, MN227661, MT829544-MT830093, and MT830095-MT830369. The paired-end reads of bacterial amplicon sequencing from the sediment samples were deposited to the National Center for Biotechnology Information of sequence read archive (SRA) under the accession number PRJNA692026.

## Results

### Taxonomic Classification of Bacterial Isolates

**FIGURE 1:**
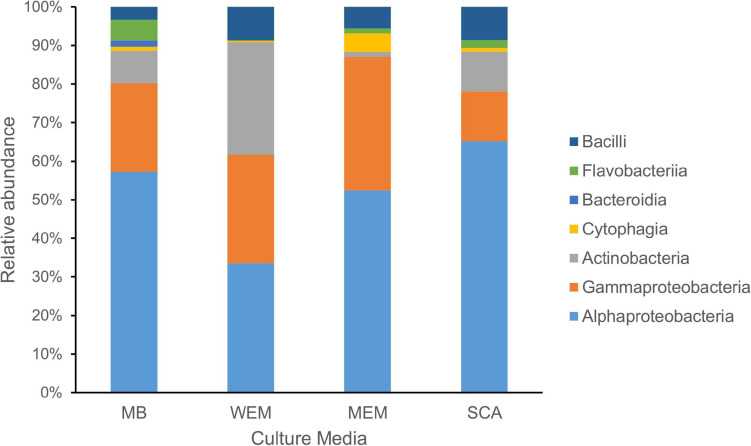
Taxonomic diversity of cultivated bacteria obtained from the coastal sediments of three habitats using four culture media. The taxonomy of isolates was assigned based on the SILVA database. MB, marine broth 2216 (299 isolates); WEM, water extracted matter medium (274 isolates); MEM, methanol extracted matter (162 isolates); SCA, starch casein agar medium (301 isolates).

More than 1,000 bacterial cultivars were isolated in pure culture from the coastal sediments of three habitats in Quanzhou Bay, China using four culture media (Supplementary Table 1 and Figure 1). The number of isolates ranged from 40 (WEM from *S. alterniflora* sediment) to 162 (MB from oyster farming sediment) after incubation for 1 month (Supplementary Table 1). Except several isolates, subcultures of these isolates grew well in MB or on MB agar plates, and 1,036 high-quality 16S rRNA gene sequences were obtained with sequence length ranging from 1,271 to 1,433 bp.

A total of 390 genotypes were observed by clustering the 1,036 isolates based on 16S rRNA gene sequence similarity of 99%. The genotype rank abundance plot showed a steep curve, which demonstrated that a few abundant genotypes were present in the culture collection, and a large number of genotypes were represented by single isolate (Supplementary Figure 2).

Based on the identification of 16S rRNA gene sequences and phylogenetic placement, the genotypes were divided into seven classes (Figure 1). Alphaproteobacteria was the most cultivated group obtained using four culture media, and representatives were present in all sediment samples of the three habitats, accounting for 52.51% of all isolates. Gammaproteobacteria and Actinobacteria accounted for 23.26 and 13.32% of the isolates using the four culture media, respectively. Firmicutes (Bacilli) and Bacteroidetes (classes Flavobacteriia, Cytophagia, and Bacteroidia) were minor cultivated groups, present in most samples.

**FIGURE 2:**
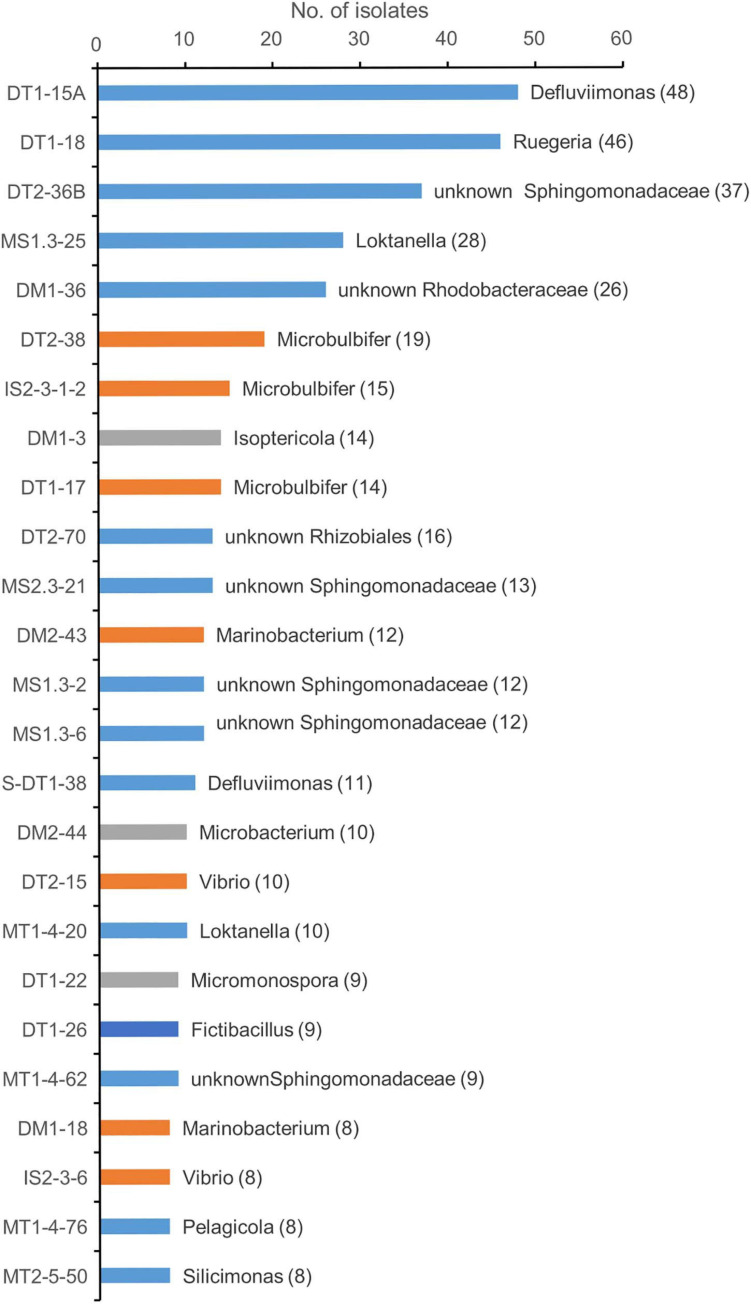
Taxonomic diversity of predominant genotypes at genus level. Different color represents the classification of the representative genotype at class ranks: blue, orange, gray, and dark blue for Alphaprotebacteria, Gammaproteobacteria, Actinobacteria, and Bacilli, respectively. The genotypes were classified using SILVA database. The representative isolate was shown. The numbers in the parentheses showed the number of genotypes.

The first two genotypes in the rank abundance curve were taxonomically classified into two genera, *Defluviimonas* and *Ruegeria*, with 48 and 46 isolates, respectively (Figure 2 and Supplementary Figure 2). Both are affiliated to the family “Rhodobacteraceae” of the order Rhodobacterales of the class Alphaproteobacteria. The third cultivated genotype (representative isolate no. DT2-36B) was affiliated to the family Sphingomonadaceae, order Sphingomonadales, based on LCA analysis, but showed close relationship with *Erythrobacter* members based on sequence similarity comparison against SILVA SSU 132 Database, RDP Database, and EzBioCloud Database. The three predominant genotypes affiliated to the Gammaproteobacteria were all taxonomically classified into the genus *Microbulbifer*, with 19, 15, and 14 isolates, respectively (Figure 2). In the Actinobacteria group, the cultivars affiliated to two genera, namely, *Isoptericola* (family Promicromonosporaceae, order Cellulomonadales) and *Microbacterium* (family Microbacteriaceae, order Microbacteriales), were found as most isolated groups, which were slightly more abundant compared to *Micromonospora* within the family Micromonosporaceae of the order Micromonosporales (Figure 2). The most cultivated isolates within the Bacilli group were taxonomically classified into the genus *Fictibacillus* (Figure 2).

### Cultivated Members Compared Among Four Culture Media

A total of 299 cultivars were isolated from three habitat sediment samples using MB medium (Supplementary Tables 1, 2). They were grouped into 153 genotypes defined at 99% sequence similarity, which showed a similar trend by using SCA medium, with a total of 301 isolates grouping into 150 genotypes (Supplementary Table 2). A total of 274 cultivars were obtained using WEM medium, grouping into 128 genotypes, whereas the smallest number of genotypes were observed using MEM medium, grouping into 69 genotypes. The number of genotypes identified from MB, WEM, and SCA was two times more than the MEM group (Supplementary Table 1), indicating that three culture media had advantages in the recovery of diverse cultivated bacteria. The Shannon diversity index was higher for the MB, WEM, and SCA groups than for MEM medium, which also supported the result (Supplementary Table 1).

Alphaproteobacteria and Gammaproteobacteria were the dominant cultivars isolated using MB medium, accounting for 57.19 and 23.08%, respectively. The two groups were also the dominant groups using MEM medium, accounting for 52.47 and 34.57%, respectively, and using SCA medium, accounting for 65.12 and 12.96%, respectively (Figure 1). Interestingly, the taxonomic affiliations of cultivars obtained using WEM showed a distinct composition compared to three culture media, which comprised of Alphaproteobacteria, Actinobacteria, and Gammaproteobacteria, accounting for 33.58, 29.20, and 28.10%, respectively (Figure 1). Obviously, more *Actinobacteria* were recovered using WEM medium compared to the three media, namely, MB, SCA, and MEM.

### Phylogenetic Diversity of Actinobacteria Recovered From Three Habitats

**FIGURE 3:**
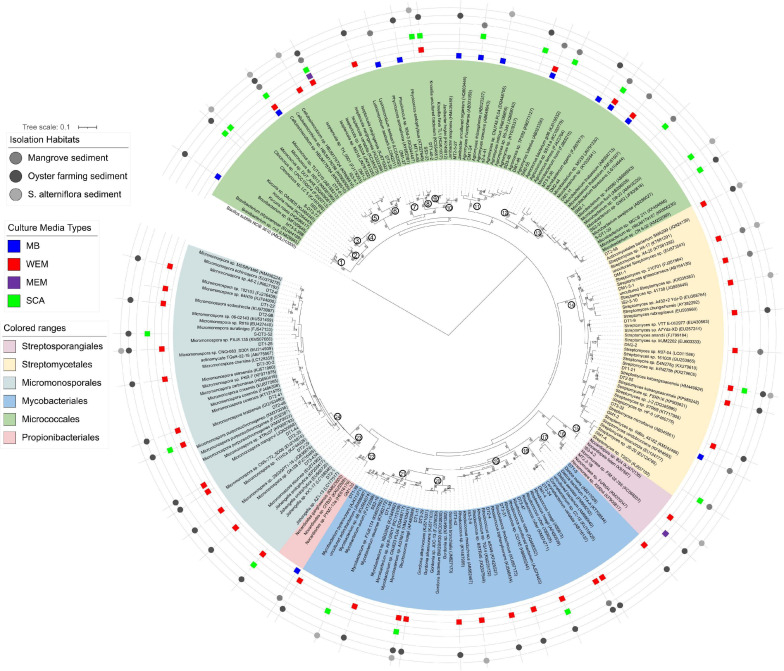
Maximum-likelihood phylogenetic tree based on the 16S rRNA gene sequences of actinobacterial isolates obtained from the three habitats using four culture media. Bootstrap values (>70) after 100 resampling are shown above the tree branches. Bar, 0.1, estimated nucleotide substitution. Numbers in the circle at the node of each clade represented the genus name: 1, *Brevibacterium*; 2, *Kocuria*; 3, *Citrococcus*; 4, *Micrococcus*; 5, *Cellulosimicrobium*; 6, *Isoptericola*; 7, *Lysinimicrobium*; 8, *Phycicoccus*; 9, *Knoellia*; 10, *Janibacter*; 11, *Agromyces*; 12, *Agrococcus*; 13, *Microbacterium*; 14, *Streptomyces*; 15, *Nocardiopsis*; 16, *Non-omuraea*; 17, *Dietzia*; 18, *Corynebacterium*; 19, *Rhodococcus*; 20, *Gordonia*; 21, *Mycobacterium*; 22, *Nocardioides*; 23, *Jishengella*; 24, *Micromonospora*.

To further interrogate the phylogenetic diversity of actinobacterial groups recovered from coastal sediments of the three habitats using four culture media, a maximum-likelihood phylogenetic tree was constructed with the reference taxa retrieved from the SILVA SSU Database (Figure 3).

A total of 138 isolates were found to belong to Actinobacteria, grouped into 63 genotypes at the threshold of 99% sequence similarity. These cultivars could be phylogenetically and taxonomically classified into 24 genera, 15 families within 6 orders, and the majority belonged to the Micrococcales, Micromonosporales, Streptomycetales, and Mycobacteriales (Figure 3 and Supplementary Table 3). The most diverse cultivars were affiliated to the genera *Micromonospora* (family Micromonosporaceae) and *Streptomyces* (family Streptomycetaceae), with 13 and 11 genotypes, respectively (Figure 3 and Supplementary Table 2).

Forty-two of the actinobacterial genotypes were obtained by using WEM medium, showing that WEM medium enables isolation of more actinobacterial groups from marine sediment than the other media used (Figure 3 and Supplementary Table 2). They were classified into 14 genera with high diversity of *Micromonospora* (12 genotypes) and *Streptomyces* (9 genotypes). SCA media also recovered 21 genotypes of actinobacteria, accounting for 14.0% of all genotypes. They were grouped into 13 genera with an average of 1.6 genotypes.

There were five genera of actinobacteria shared by WEM and SCA, *Rhodococcus*, *Micromonospora*, *Mycobacterium*, *Isoptericola*, and *Knoellia*. *Cellulosimicrobium*, *Corynebacterium*, *Dietzia*, and *Gordonia* were only recovered in the WEM group. *Agromyces*, *Citricoccus*, *Kocuria*, *Microbacterium*, and *Micrococcus* were only recovered in the SCA group (Figure 3 and Supplementary Figure 3). *Micromonospora* were mostly isolated from oyster farming sediments using WEM, and they were less recovered in *S. alterniflora* and mangrove sediments. Only two genotypes were observed in the MEM group. This result supported that WEM could enhance the recovery of more diverse actinobacteria compared to the other culture media.

### Bacterial Community Recovered Using 16S rRNA Gene Amplicon Sequencing

**FIGURE 4:**
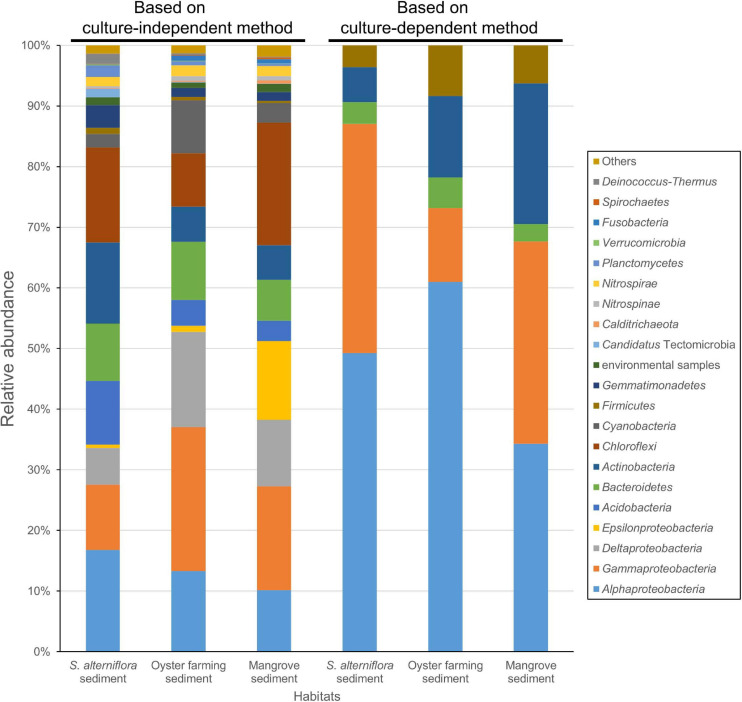
Bacterial groups for the coastal sediments of the three habitats determined by using 16S rRNA gene amplicon sequencing (left three columns) and by cultivation (right three columns). The phylum Proteobacteria is shown at the class level, that is Alphaproteobacteria, Gammaproteobacteria, Deltaproteobacteria, and Epsilonproteobacteria. The taxonomy of ASVs from each sample was assigned based on the lowest common ancestor (LCA) method.

Amplicon sequencing of the bacterial 16S rRNA gene V3–V4 fragment revealed that 1,083–1,512 ASVs were identified from the nine *S. alterniflora* sediment samples, with the largest tag number (28,169 tags) of sample 19S2.2 and the smallest tag number (9,203 tags) of sample 20S3.2 (Supplementary Table 4). They were taxonomically classified into seven dominant bacterial groups at the phylum or class level: Chloroflexi (16.0 ± 6.0%), Alphaproteobacteria (14.7 ± 6.2%), Gammaproteobacteria (13.2 ± 6.8%), Actinobacteria (11.3 ± 6.1%), Acidobacteria (10.0 ± 7.9%), Deltaproteobacteria (8.6 ± 7.9%), and Bacteroidetes (8.4 ± 3.8%) (Figure 4 and Supplementary Figure 4).

**FIGURE 5:**
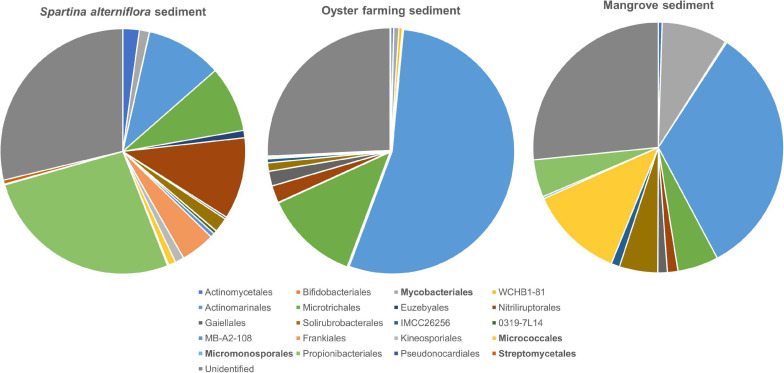
Taxonomic classification and relative abundance of *Actinobacteria* at the order level identified from the three habitats. The numbers of ASVs belonging to Actinobacteria found in *S. alterniflora* sediment, oyster farming sediment, and mangrove sediment are 511, 329, and 195, respectively. The taxonomy was assigned based on the SILVA database. The four orders detected by cultivations are marked bold.

A total of 511 ASVs affiliated to the Actinobacteria were identified in *S. alterniflora* sediments, and they could be classified into nine dominant orders (>1%), consisting of Propionibacteriales (accounting for 26.53%), Nitriliruptorales (10.78%), “*Candidatus* Actinomarinales” (10.03%), “*Candidatus* Microtrichales” (8.70%), Frankiales (4.50%), Actinomycetales (2.17%), Solirubrobacterales (1.93%), Mycobacteriales (1.32%), and Kineosporiales (1.19%) (Figure 5). By mapping the actinobacterial ASVs to the nearly full-length 16S rRNA gene sequences of the isolates of Actinobacteria using blast+ program, only 2.94% of actinobacterial ASVs had 99.0–100% sequence similarity with the isolates. The mapped Actinobacteria isolates belonged to the four orders: Micrococcales (two genotypes), Streptomycetales (one genotype), Mycobacteriales (two genotypes), and Micromonosporales (one genotype).

Amplicon sequencing of the 16S rRNA gene V3–V4 fragment revealed 1,059–1,887 ASVs (1,504 ASVs in average) from the 18 oyster farming sediment samples with the largest number (24,293 tags) of sample 19MT1.1 and the smallest number (9,059 tags) of sample 20MT3.3 (Supplementary Table 4). There were seven dominant bacterial groups classified at the phylum or class level: Gammaproteobacteria (24.3 ± 3.7%), Deltaproteobacteria (15.7 ± 2.6%), Alphaproteobacteria (12.67 ± 4.7%), Chloroflexi (9.7 ± 4.5%), Bacteroidetes (8.6 ± 4.2%), Cyanobacteria (8.5 ± 4.6%), and Actinobacteria (5.8 ± 1.7%) (Figure 4 and Supplementary Figure 4). In Gammaproteobacteria, Woeseiaceae was found as a dominant and constant group in the sediment of oyster farming habitat, accounting for 8.65% (±1.08%) of the total bacteria (Supplementary Figure 5). The other dominant groups classified at the family level belonged to Kiloniellaceae and Rhodobacteraceae in Alphaproteobacteria, Desulfobacteraceae and Desulfobulbaceae in Deltaproteobacteria, and Anaerolineaceae in the phylum Chloroflexi (Supplementary Figure 5).

A total of 329 ASVs belonging to the phylum Actinobacteria were identified, and they could be classified into 2 dominant orders, namely, “*Candidatus* Actinomarinales” and “*Candidatus* Microtrichales,” accounting for 54.19 and 12.51% of the total actinobacteria, respectively. Nitriliruptorales, Gaiellales, and Solirubrobacterales accounted for 2.23, 1.93, and 1.11% of total actinobacteria, respectively. Actinomycetales, Bifidobacteriales, Mycobacteriales, Frankiales, and Micrococcales were found as minor groups, accounting for <1% (Figure 5). By mapping the ASVs to the nearly full-length 16S rRNA gene of the isolates, none of the actinobacterial ASVs had 99.0%–100% sequence similarity with the cultivated isolates, indicating that the majority of actinobacteria remains uncultivated.

Amplicon sequencing of the 16S rRNA gene V3–V4 fragment revealed 992–1,433 ASVs (1,166 ASVs in average) were classified from the five mangrove sediments with the largest number (15,058 tags) of sample 20GM.C1 and the smallest number (10,169 tags) of sample 20GM.F2 (Supplementary Table 4). There were seven dominant bacterial groups at the phylum or class level: Chloroflexi (20.0 ± 8.2%), Gammaproteobacteria (17.2 ± 3.9%), Epsilonproteobacteria (13.0 ± 8.7%), Deltaproteobacteria (11.0 ± 1.8%), Alphaproteobacteria (10.2 ± 2.3%), Bacteroidetes (6.9 ± 2.2%), and Actinobacteria (5.7 ± 3.0%) (Figure 4).

A total of 195 actinobacterial ASVs were identified in mangrove sediments, and they were taxonomically classified into six dominant orders, including “*Candidatus* Actinomarinales” as major component (accounting for 33.02%) and Micrococcales (12.18%), Mycobacteriales (8.51%), “*Candidatus* Microtrichales” (5.27%), Solirubrobacterales (4.93%), and Propionibacteriales (4.84%). Nitriliruptorales, Gaiellales, and IMCC26256 accounted for 1.36, 1.23, and 1.11% of the total actinobacteria. The cultivated members affiliated to Actinomycetales and Micromonosporales were also found as minor groups (Figure 5). By mapping the ASVs to the 16S rRNA gene sequences of the isolates, 4.62% of the actinobacterial ASVs had 99.0–100% sequence similarity with the cultivated isolates, which also proved that the majority of actinobacteria remains uncultivated. The mapped Actinobacteria isolates belonged to the two orders, namely Micrococcales (seven genotypes) and Mycobacteriales (one genotypes).

### Phylogenetic Novelty of the Isolates

The nearly full length of 16S rRNA genes of the representative isolates was subjected to sequence similarity comparison to the SILVA SSU Database, EzBioCloud Database, and RDP Database, which include sequences of both cultured and uncultivated ones with full length and high quality. The majority of the representative isolates (more than 95% isolates) was found to have >97.0% sequence similarities with previously deposited sequences in these databases (Supplementary Tables 2, 5). However, 4.05% of the representative isolates could be considered to represent a new species, based on the recommended threshold of 97.0% sequence similarity with type strains (Supplementary Table 5).

Among the isolates with low sequence similarity, four representative isolates, named SM2-F, GM2-3-6-6, SM2-16, and S-DT1-15, were found to have 16S rRNA gene sequence similarity of <94.5% with type strains, which is below the recommended genus threshold (Yarza et al., 2014). Three isolates were taxonomically classified into the Flavobacteriaceae within the class Flavobacteria and another isolate was classified into the Alphaproteobacteria (Supplementary Tables 2, 5).

## Discussion

This still ongoing study aimed to collect cultivated bacteria from coastal sediments and confirmed their taxonomic classification and also tried to isolate novel bacterial species. In addition to the MB and SCA media that are frequently used in bacterial isolation from marine environments, we used two rarely used culture media, WEM and MEM, in an attempt to expand the diversity of cultivated bacteria from coastal sediments. Because of the convenience of growth and storage of subcultures, traditional MB was used for further streaking to pure culture and preservation.

A large collection of cultures was obtained from coastal sediments of three distinct habitats in this study (Supplementary Table 1). The isolates were taxonomically classified into three major groups, namely, Alphaproteobacteria, Gammaproteobacteria, and Actinobacteria (Figure 1), which represented a bacterial composition similar to that isolated globally from offshore seawater (Joint et al., 2010) and open ocean seawater (Sanz-Sáez et al., 2020). Three strains isolated from oyster farming sediments were classified into the genera *Thauera* and *Hydrogenophaga* of Betaproteobacteriales, which are placed into an order within the class Gammaproteobacteria in SILVA SSU release 132 (Quast et al., 2013). We did not recover any isolates affiliated to the Deltaproteobacteria, Epsilonproteobacteria, and Woeseiaceae members of Gammaproteobacteria, Chloroflexi, Gemmatimonadetes, and Acidobacteria, as revealed by amplicon sequencing from these coastal sediments (Figure 4 and Supplementary Figure 5), which are widely distributed bacterial groups in coastal sediments (Mussmann et al., 2017; Hoffmann et al., 2020). Compared to community composition, the most isolated cultivars, such as *Defluviimonas*, *Loktanella*, and *Ruegeria* (Alphaproteobacteria) or *Microbulbifer* and *Marinobacterium* (Gammproteobacteria), accounted for a minor percentage (Figure 2 and Supplementary Figure 2), indicating that the majority of bacteria in coastal sediments remains uncultivated.

Except the sample of oyster farming sediment using WEM, Alphaproteobacteria and Gammaproteobacteria are the most cultivated groups in *S. alterniflora* sediment and oyster farming sediment that developed on each of the media used (Figure 1). *Defluviimonas* (Alphaproteobacteria), with representative isolate DT1-15A (48 isolates), shared maximum sequence similarity of 99.18% with *Defluviimonas aestuarii* BS14^T^, isolated from a tidal flat (Math et al., 2013). Members of *Defluviimonas* are found in coastal sediments, with type species isolated from sewage, where they possibly play important roles in nitrogen cycling (Foesel et al., 2011). The *Defluviimonas* recovered from *S. alterniflora* sediment possibly has been enriched due to the sewage effluent into this *S. alterniflora* sediment (Supplementary Figure 1). Another group of Alphaprotebacteria is *Erythrobacter* (Figure 2), which is also found as a most cultivated group in open ocean water (Sanz-Sáez et al., 2020). Our data supported that *Erythrobacter* are generalists in the marine environment from coastal sediment to the open ocean.

*Alteromonas* (Gammproteobacteria) accounted for a small proportion of the isolates recovered from three habitats (Figure 2). However, a recent study found that *Alteromonas* was the most isolated member using traditional MB medium in open ocean water (Sanz-Sáez et al., 2020). By contrast, *Microbulbifer* species are found as most cultivated members recovered from coastal sediments (Figure 2 and Supplementary Table 2). The representative species (isolate DT1-17) has sequence similarity of 99.86% with *Microbulbifer mangrovi* DD-13^T^, which also has the ability of degrading more than 10 polysaccharides, including agar, alginate, chitin, cellulose, starch, and carrageenan (Vashist et al., 2013). An agar-eating isolate IS2-3-1-2 is close to *Microbulbifer hydrolyticus* IRE-31^T^, which was found to produce various hydrolytic enzymes for degradation of cellulose, xylan, chitin, and gelatin (Gonzalez et al., 1997). Thus, we speculate that *Microbulbifer* could be important organic matter degraders in coastal sediments. In view of marine enzyme resource development, these *Microbulbifer* species with a complement of enzymes could be considered as important bacteria for the utilization of enzymes in future applications.

Using media with soil extract by 80% methanol as sole carbon source was reported as an effective method to isolate novel uncultured bacteria from soil (Nguyen et al., 2018). However, the MEM method did not demonstrate its potential in recovery novel bacterial taxa and improvement of the species diversity from marine sediment. Additionally, only two cultivars, namely, MT3.3-3A and MT2.3-13A, were found to represent novel species, which had the maximum 16S rRNA gene sequence similarity of 96.8% with *Reinekea marinisedimentorum* DSM 15388^T^ and *Actibacterium ureilyticum* LS-811^T^ (Supplementary Table 5).

Marine actinobacteria are gaining more and more attention (Bull et al., 2005; Bull and Stach, 2007) due to their capacity of producing various natural products, such as antibiotics (Manivasagan et al., 2013; Manivasagan et al., 2014). Amplicon sequencing revealed a high relative abundance of uncultivated actinobacterial orders (Figure 5). “*Candidatus* Actinomarinales,” reported exclusively in surface seawater (Lopez-Perez et al., 2020), was found to be a dominant actinobacterial member in coastal sediments (Figure 5), which made us question their geographical distribution. Sequence comparison of 16S rRNA gene found that the ASVs affiliated to “*Candidatus* Actinomarinales” had <86.9% similarity with the representative species “*Candidatus* Actinomarina minuta” (accession number KC811136), a marine actinobacteria with low G + C content and ultrasmall genome (Ghai et al., 2013). Another dominant actinobacteria in the three habitats was “*Candidatus* Microtrichales,” which remain uncultivated thus far, hindering our understanding of its metabolic and ecological function. Nitriliruptorales were found as more dominant in *S. alterniflora* sediment than in oyster farming sediment and mangrove sediment. Until now, there was only one species, *Nitriliruptor alkaliphilus* (Sorokin et al., 2009), described in this order that was isolated from soda lake sediment and grown on the nitrile compounds as sole carbon source.

Although the cultivated actinobacterial isolates accounted only for a minor group (<5%), we still obtain a large number of isolates of actinobacteria, which were grouped into 6 orders spanning 24 genera and 15 families especially from oyster farming sediment and mangrove sediment (Figure 3). We found that WEM enhances the recovery of diverse actinobacterial species from coastal sediments compared to SCA, which was frequently used for isolation of marine actinomycetes species^1^ (Bukhari et al., 2013; Mohseni et al., 2013). Diverse *Micromonospora* (11 representative genotypes) were recovered from oyster farming sediment. Considering the diverse secondary metabolites produced by *Micromonospora* (Subramani and Aalbersberg, 2013; Qi et al., 2020), this culture method could be an effective and simple method for the recovery of this genus. WEM also recovered the majority of isolates belonging to *Streptomyces* (Figure 3). *Streptomyces* spp. are also promising producers of antibiotics (Subramani and Aalbersberg, 2013).

Culture media usually employed for bacterial growth in laboratory are nutritionally rich, containing peptone and yeast extract, etc., while it is reported that much-low-nutrient medium could yield diverse bacteria (Connon and Giovannoni, 2002). Thus, one hypothesis regarding the enhancement of cultivability of actinobacteria was that WEM medium is possibly a nutritionally poor medium that included low nutrients extracted from sediment, which are more appropriate for the growth of culturable actinobacteria. Another hypothesis was that more low-molecular-weight organic substances may be included in the WEM medium, such as acetoacetic acid and oxaloacetic acid, which are reported on soil extract (Nguyen et al., 2018). The low-molecular-weight organic substances may selectively support the growth of culturable actinobacteria. In sum, our study supports the idea that coastal sediments are a natural microbial reservoir for actinobacteria congruent with previous studies (Jose and Jha, 2017). Pretreatment of sediment samples used for the preparation of the WEM medium by heating or drying could be tested in the future in an attempt to further improve the recovery of more diverse actinobacteria (Subramani and Aalbersberg, 2013; Terahara et al., 2013).

Four culture media could recover a certain number of novel bacteria (∼4.0%) from the coastal sediments, reflecting the fact that marine coastal sediments are rich reservoirs of novel bacterial resources. Although only a small proportion of the isolates belonged to the Bacteroidetes (Figure 1), several potentially novel species were identified from this group. The 16S rRNA gene sequences of the novel isolates were affiliated to the family of Flavobacteraceae but had low similarities with species with validly published names (Supplementary Table 5).

## Conclusion

Getting bacteria from natural environments into cultures in laboratory is still a compelling need for microbial studies, together with the characterization of the majority of uncultivated bacteria revealed by molecular methods. As culture media are always biased toward the recovery of certain types of culturable bacteria only, we recommend that different culture media be combined to investigate the cultivated bacteria in a given habitat. Aqueous extraction of sediments as sole nutrient source may enhance recovery of bacteria from coastal sediments, especially for the recovery of rare actinobacteria. In addition, our study has yielded a large culture collection for further genomic analysis and elucidation of the physiological and ecological function of the marine bacteria.

## Data Availability Statement

The datasets presented in this study can be found in online repositories. The names of the repository/repositories and accession number(s) can be found in the article/Supplementary Material.

## Author Contributions

ZH conceived the design of the project and wrote the manuscript. All authors conducted the experiment.

## Conflict of Interest

The authors declare that the research was conducted in the absence of any commercial or financial relationships that could be construed as a potential conflict of interest.

## Footnotes

1 https://microbiologyinfo.com/

## Supplementary Material

Supplementary Figure 1 Maps showing the samples collected in this study.

Supplementary Figure 2 Rank abundance curve showing relationship of representative genotypes (99% clustering) with the number of isolates.

Supplementary Figure 3 Venn diagram showing overlapped actinobacterial genera obtained by using three culture media. MB, marine broth 2216; WEM, water extracted matter medium; SCA, starch casein agar medium.

Supplementary Figure 4 Bacterial community composition of the coastal sediments from the three habitats. The phylum *Proteobacteria* is shown at the class level, that is *Alphaproteobacteria*, *Gammaproteobacteria*, *Deltaproteobacteria* and *Epsilonproteobacteria*. The taxonomy of ASVs from each sample was assigned based on the lowest common ancestor (LCA) method. The green, blue and red boxes represented the three coastal habitats, *S. alterniflora* sediment (9 samples), oyster farming sediment (18 samples) and mangrove sediment (5 samples), respectively.

Supplementary Figure 5 Bacterial community composition of the coastal sediment samples classified at the family level.

Supplementary Table 1 Sampling information and data statistics for culturable bacteria.

Supplementary Table 2 Classification of the isolates obtained using four culture media. The isolates represented novel species or novel genera (having < 97% 16S rRNA gene sequence similarities with validly published species) are marked red.

Supplementary Table 3 Classification of representative genotypes in *Actinobacteria*.

Supplementary Table 4 Sample description and statistics of 16S rRNA gene V3-V4 amplicon sequencing.

Supplementary Table 5 Taxonomy and sequence similarity of the isolates representing novel species.
