# Supplementary figures and images for: A Simple Culture Method Enhances the Recovery of Culturable Actinobacteria From Coastal Sediments
Source: Front Microbiol. 2021 Jun 14;12:675048. doi: 10.3389/fmicb.2021.675048 (PMC8236954; doi:10.3389/fmicb.2021.675048)

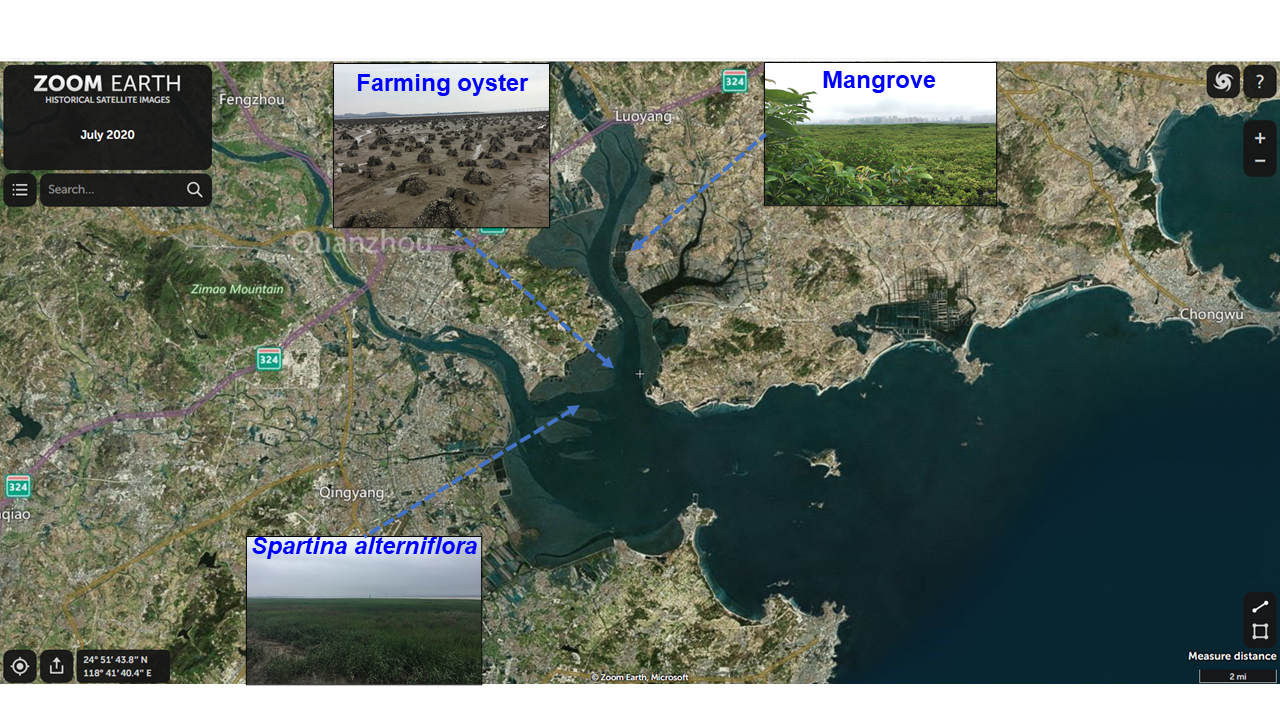

Supplement: Supplementary Figure 1 — Maps showing the samples collected in this study. [file Image_1.TIF]

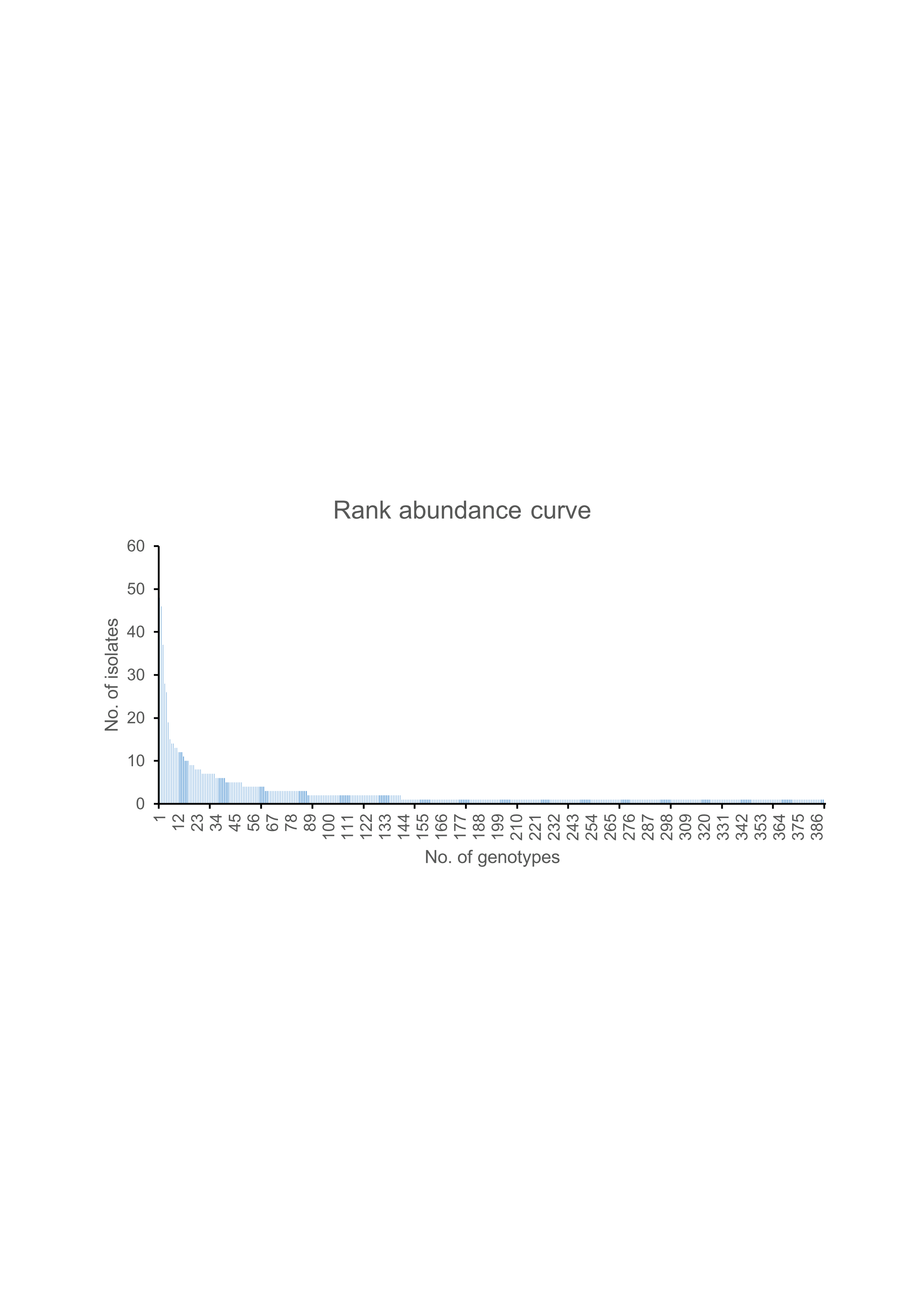

Supplement: Supplementary Figure 2 — Rank abundance curve showing relationship of representative genotypes (99% clustering) with the number of isolates. [file Image_2.TIF]

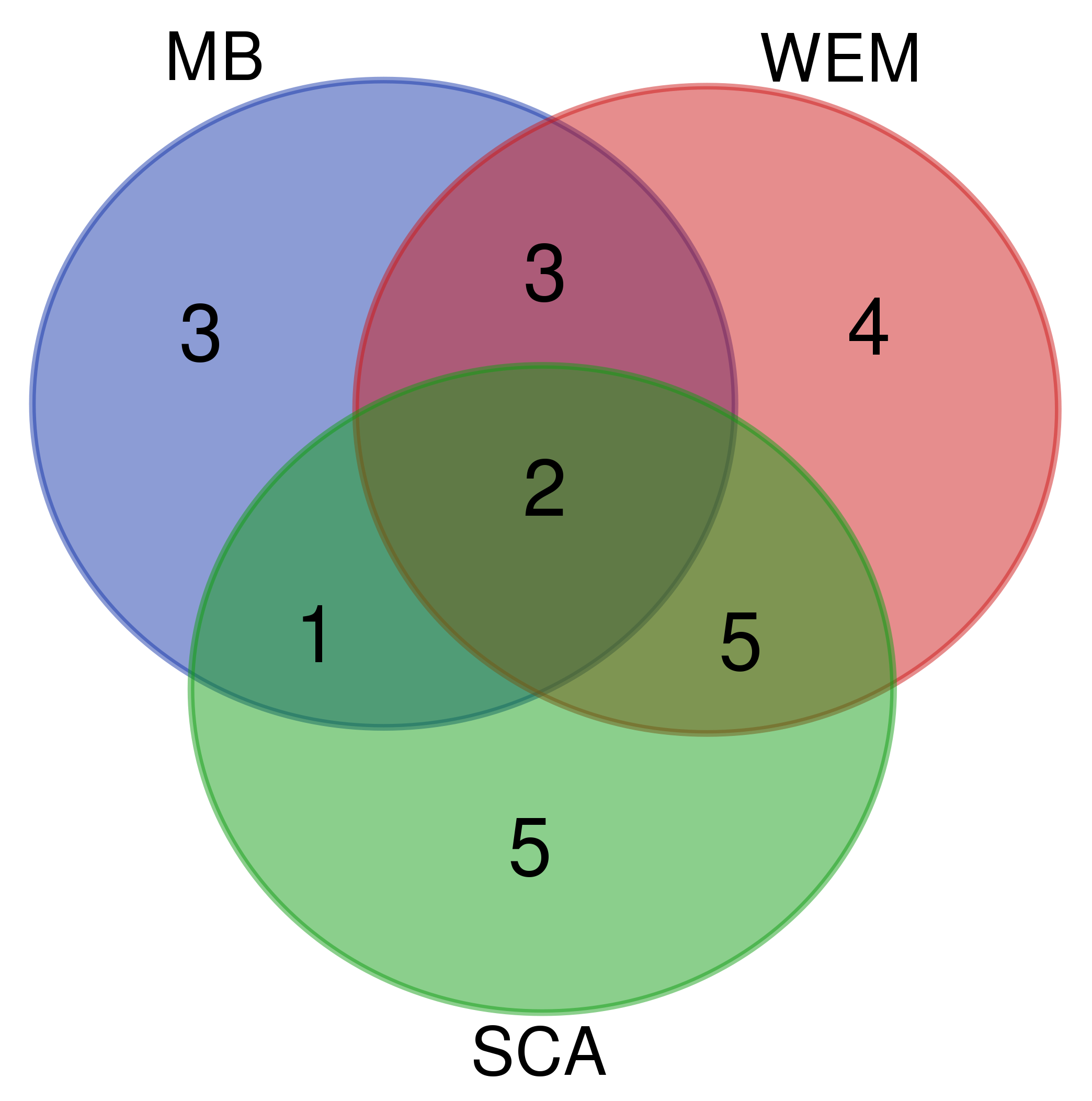

Supplement: Supplementary Figure 3 — Venn diagram showing overlapped actinobacterial genera obtained by using three culture media. MB, marine broth 2216; WEM, water extracted matter medium; SCA, starch casein agar medium. [file Image_3.PNG]

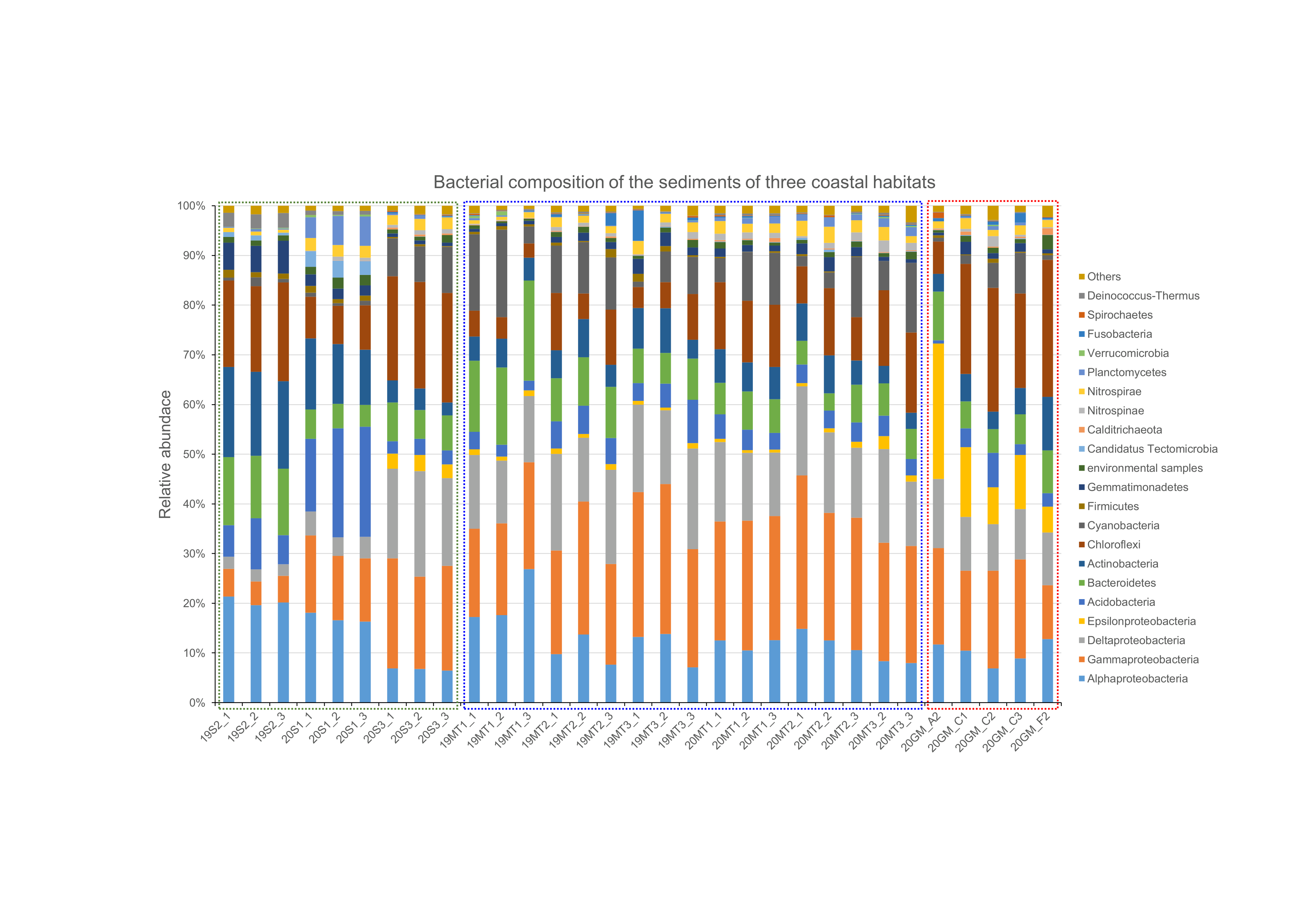

Supplement: Supplementary Figure 4 — Bacterial community composition of the coastal sediments from the three habitats. The phylum Proteobacteria is shown at the class level, that is Alphaproteobacteria, Gammaproteobacteria, Deltaproteobacteria and Epsilonproteobacteria. The taxonomy of ASVs from each sample was assigned based on the lowest common ancestor (LCA) method. The green, blue and red boxes represented the three coastal habitats, S. alterniflora sediment (9 samples), oyster farming sediment (18 samples) and mangrove sediment (5 samples), respectively. [file Image_4.TIF]

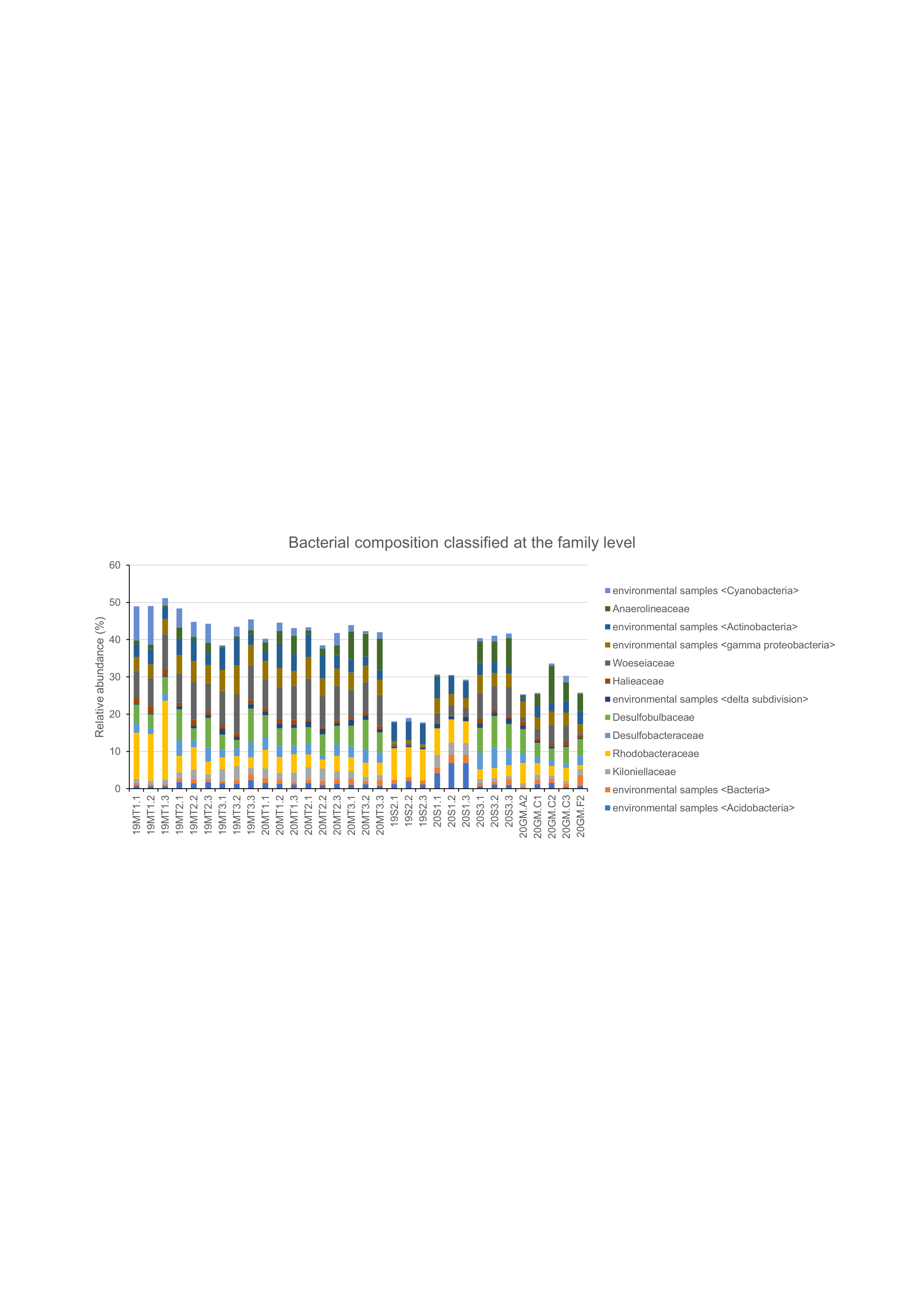

Supplement: Supplementary Figure 5 — Bacterial community composition of the coastal sediment samples classified at the family level. [file Image_5.TIF]
